# Investigating the effect of *Fusobacterium nucleatum* on the aggressive behavior of cancer-associated fibroblasts in colorectal cancer

**DOI:** 10.1007/s12672-024-01156-0

**Published:** 2024-07-19

**Authors:** Fatemeh Eskandari-Malayeri, Marzieh Rezeai, Tahmineh Narimani, Nafiseh Esmaeil, Mahdieh Azizi

**Affiliations:** 1 Department of Immunology, School of Medicine, Isfahan University of Medical Sciences, Isfahan, Iran; 2 Department of Microbiology, School of Medicine, Isfahan University of Medical Sciences, Isfahan, Iran

**Keywords:** Colorectal cancer, *Fusobacterium nucleatum*, Cancer-Associated fibroblasts (CAF), Fibroblast activation protein (FAP), α-Smooth muscle actin (α-SMA), IL-6, TGF-β

## Abstract

*Fusobacterium nucleatum*, (*F. nucleatum*) as a known factor in inducing oncogenic, invasive, and inflammatory responses, can lead to an increase in the incidence and progression of colorectal cancer (CRC). Cancer-associated fibroblasts (CAF) are also one of the key components of the tumor microenvironment (TME), which lead to resistance to treatment, metastasis, and disease recurrence with their markers, secretions, and functions. This study aimed to investigate the effect of *F. nucleatum* on the invasive phenotype and function of fibroblast cells isolated from normal and cancerous colorectal tissue. *F. nucleatum* bacteria were isolated from deep periodontal pockets and confirmed by various tests. CAF cells from tumor tissue and normal fibroblasts (NF) from a distance of 10 cm of tumor tissue were isolated from 5 patients by the explant method and were exposed to secretions and ghosts of *F. nucleatum*. The expression level of two markers, fibroblast activation protein (FAP), and α-smooth muscle actin (α-SMA), and the amount of production of two cytokines TGF-β and IL-6 from fibroblast cells were measured by flow cytometry and ELISA test, respectively before and after exposure to different bacterial components. The expression of the FAP marker was significantly higher in CAF cells compared to NF cells (P < 0.05). Also, the expression of IL-6 in CAF cells was higher than that of NF cells. In investigating the effect of bacterial components on the function of fibroblastic cells, after comparing the amount of IL-6 produced between the normal tissue of each patient and his tumoral tissue under 4 treated conditions, it was found that the amount of IL-6 production from the CAF cells of patients in the control group, treated with heat-killed ghosts and treated with paraformaldehyde-fixed ghosts had a significant increase compared to NF cells (P < 0.05). Due to the significant increase in FAP marker expression in fibroblast cells of tumor tissue compared to normal tissue, it seems that FAP can be used as a very good therapeutic marker, especially in patients with high levels of CAF cells. Various components of *F. nucleatum* could affect fibroblast cells differentially and at least part of the effect of this bacterium in the TME is mediated by CAF cells.

## Introduction

Colorectal cancer (CRC) is the third most common cancer and the fourth leading cause of cancer death in the world [1–3]. Despite the existence of systemic treatments such as chemotherapy and radiotherapy, a large number of patients are facing disease progression due to resistance to treatment. Accordingly, the 5-year survival is less than 10% in CRC patients who are in stage IV of the disease [4]. The rapid progress and resistance to existing treatments have highlighted the need to carefully investigate the cause of CRC development, progress, and resistance to treatment.

Tumor cells can survive and progress due to place in a dynamic and complex environment including fibroblasts, endothelial cells, immune cells, microbiota, as well as extracellular matrix components such as collagen, fibronectin, and hyaluronan, which is collectively called the tumor microenvironment (TME) [5].

Cancer-associated fibroblast (CAF) is one of the main components in TME that plays a critical role in communicating and maintaining the support network for tumor cell [6]. These cells have complex origins such as epithelial or endothelial cells, cancer stem cells, and adipocytes, but mainly derived from normal fibroblasts (NF) under tumor inflammatory conditions [7]. The presence of activated CAFs as the prominent components in solid tumors, such as pancreatic adenocarcinoma, intestinal, and breast cancer is associated with poor prognosis and resistance to treatment, metastasis, and disease recurrence [8, 9].

The activation state, stress response, tumor stages, and origin of CAFs cause functional heterogeneity [10, 11]. So various studies have proved the tumor-promoting and suppressive functions of CAFs [12, 13]. CAF promotes cancer progression through oncogenic functions, including the secretion of many different growth factors and proinflammatory cytokines, such as transforming growth factor beta (TGF-β), interleukin 6 (IL-6), vascular endothelial growth factor (VEGF), and C-X-C motif chemokine 12 (CXCL-12) [14–18]. Also, their role in metastasis, therapeutic resistance, regulation of angiogenesis, metabolism, immune cell modulation, and expression or induction of inhibitory checkpoints, such as programmed cell death-1 (PD-1) and cytotoxic T-lymphocyte-associated antigen-4 (CTLA-4) have been indicated in previous studies [8, 19–22]. On the contrary, the tumor-suppressive role of CAFs has been reported recently. For instance, the deletion of α-SMA + myofibroblasts in pancreatic ductal adenocarcinoma (PDAC) suppresses immune surveillance by increasing CD4^+^Foxp3^+^ regulatory T cells (Tregs) in TME [23].

The heterogeneity of CAFs is not only reflected in their origin and function but can also be identified by diversity in markers. Based on different markers, they perform different functions [10]. Several markers have been used to confirm the presence of CAFs. Among these, some markers, including α-smooth muscle actin (α-SMA), platelet-derived growth factor receptor (PDGFR) α/β, podoplanin (PDPN), fibroblast activation protein (FAP), and fibroblast specific protein 1 (FSP1) are introduced as important markers in the diagnosis and targeting of CAFs [24–26].

FAP is a type II integral serine protease that could be mentioned as one of the most important markers in the stroma of CAFs rich solid tumors in so far as several studies have used it as a marker of CAFs [10, 27]. FAP-expressing CAF cells could mediate immunosuppression, as removal of these cells enhanced T cell mediated killing of tumor cells [28]. Also, FAP-expressing CAFs upregulate the expression of proinflammatory genes and may be responsible for tumor immune evasion in a mouse model of PDAC [21]. In addition to the effect of FAP on the immune system, this marker plays an essential role in tumor growth and development, angiogenesis, metastasis, extracellular matrix regeneration, and immune system suppression [29, 30].

α-SMA, another important marker to identify CAF populations, induces the secretion of cytokines such as CXCL-12 and IL-6 and extracellular matrix (ECM) remodeling [31]. It is also associated with a higher risk of recurrence and shorter overall survival in patients with CRC as a prognostic factor [32–36].

The interaction of cancer cells with microbiota is a unique feature in CRC compared to other cancers [4]. In a healthy intestine, microbiota plays a pivotal role in human physiological and natural activities, including digestion, metabolism, immune homeostasis, and growth of intestinal lymphatic tissues [37]. The change in the balance of commensal bacteria following various conditions leads to the reduction of the diversity of microbial populations and the predominance of opportunistic pathogenic bacteria such as *Fusobacterium nucleatum*, (*F. nucleatum*) and *Enterococcus faecalis (E. faecalis)* [38]. The increase of these pathogens leads to the production of harmful metabolites, including various toxins and antigens that cause maladaptive immune responses and inflammation, DNA damage, genomic instability, and ultimately the initiation and progression of tumors [39]. In this regard, metagenomics analyses in patients with CRC show the significant presence of *F. nucleatum* in their intestines and feces. *F. nucleatum* is a gram-negative anaerobic bacteria that as an opportunistic pathogen, is closely related to CRC, and the progression of adenoma to CRC and its increased presence in tumor tissues is associated with a poor prognosis for patients [40]. *F. nucleatum* leads to the production of inflammatory cytokines such as interleukin-1 beta (IL-1β), interleukin-8 (IL-8), and IL-6 by activating the nuclear factor NF-κB and it can change the conditions in favor of CRC development and progression [41, 42].

Despite extensive studies on the role of CAFs and *F. nucleatum* in the CRC environment, there are very few studies addressing the interactions between these two key components in the microenvironment of CRC. In this regard, the aim of the present study is a preliminary investigation regarding the effect of *F. nucleatum* on the function and phenotype of fibroblast cells isolated from the tumor environment and the adjacent normal tissue.

## Materials and methods

### Bacterial isolation and identification

*F. nucleatum* was isolated from the periodontal tooth pockets in patients with chronic periodontal pockets who were referred to the Department of Periodontology, Faculty of Dentistry, Isfahan University of Medical Sciences between December 2020 and July 2021. Patients had taken antibiotics for at least one month before the sampling and had a pocket depth ≥ 5 mm. To remove normal oral flora, the surface of dental abscesses was wiped off with sterile gas and 70% ethyl alcohol. Then a paper point (size 40) was inserted deeply into the patient's periodontal pockets and held for 30 s. The samples were streaked on Columbia agar (CA) (CM0331, Oxoid, UK) supplemented with 5 µg/ml hemin, 10 µg/ml vitamin K, 5% defibrinated sheep blood, 1 μg/ml norfloxacin (Sigma, USA), and 4 μg/ml vancomycin (Sigma, USA) and placed in an anaerobic jar containing type A GasPak (Anaerocult A, Merck KGaA, Germany) to create anaerobic conditions, and were transferred to the laboratory [43].

Cultures incubated at 37 ºC for 48 h and sub-cultured several times on CA supplemented with 5 µg/ml hemin, 10 µg/ml vitamin K, and 5% defibrinated sheep blood, 1 μg/ml norfloxacin, and 4 μg/ml vancomycin under anaerobic conditions (N2:CO2:H2, 85∶5∶10). The growth on the plate was examined macroscopically and samples with colonies similar to *F. nucleatum* were gram-stained, and subsequently, biochemical tests were performed to confirm bacterial identity. In this regard, antibiotic sensitivity was carried out for colistin (10 µg/ml), penicillin (10 µg/ml), kanamycin (1000 µg/ml), and vancomycin (5 µg/ml) using a CA plate without antibiotic, hemin, and vitamin K1. The plates were placed in an anaerobic jar at 37 ºC for 48 h and then checked visually for the presence or absence of the zone of inhibition. To perform the indole and lipase test, the isolates were cultured on Sulfide Indole Motility (SIM) (CM0435, Oxoid, UK) and Egg Yolk agar (EYA) (CM0131, Oxoid, UK) plate, respectively, and were incubated in an anaerobic jar at 37 ºC for 48 h.

Finally, the identified isolates were stored in brain heart infusion broth (BHI) (CM1135, Oxoid, UK) at –80 °C.

### Bacterial ghosts’ and secretome preparation

For preparation of the soluble fraction from *F. nucleatum*, preserved bacteria were reconstituted in 100 ml BHI broth with 150 × 10^6^ bacteria corresponding to 0.5 McFarland in anaerobic condition for 48 h. After centrifugation of the cultured medium, the supernatant was filtered through the 0.22 µm sterile filter and stored at −70. For the preparation of bacterial ghosts, the pellet was washed three times and dissolved in PBS and the concentration was determined at 600 nm. The prepared bacterial suspension was submitted to kill heat and Paraformaldehyde (PFA), respectively. For heat killing, 2 ml of bacterial suspension in PBS was subjected to a temperature of 100 °C for 10 min and stored at −70 °C. For PFA fixation, 2 ml of bacterial suspension in PBS was centrifuged and the bacterial pellet dissolved in 4 ml of 1% PFA. The solution was incubated for 30 min at room temperature in the dark. Then the solution was centrifuged, the ghost was washed twice, dissolved in 2 ml of PBS, and stored at −70.

### Isolation of normal and *cancer*-associated fibroblasts (CAFs)

To isolate fibroblast cells, 20 patients with CRC who underwent surgery from September 2021 to April 2022 at the Alzahra Hospital (Isfahan, Islamic Republic of Iran), were included in the study. Patients, who were selected for sampling, had not undergone chemotherapy, radiotherapy, or immunotherapy before surgery and had no history of autoimmune diseases. Sampling was done in the operating room by a surgeon under the supervision of a pathologist to select CRC tissue and adjacent normal tissue at a distance of 10 cm from the tumor. Due to the limited life span of the isolated primary cells or contamination with bacteria or fungi, only samples from 5 patients [CAFs and normal fibroblasts (NF)] were obtained. Demographic characteristics of included subjects are summarized in (Table 1).

**Table 1:** Demographics characteristics of the patients with colorectal cancer

Patient	Sex	Age	Tumor location	Tumor size	Histological type	TNM stage
1	Male	58	Rectosigmoid	4 cm	Adenocarcinoma	Stage III
2	Male	82	Rectosigmoid	3 cm	Adenocarcinoma	Stage II
3	Male	80	Sigmoid	3 cm	Adenocarcinoma	Stage III
4	Female	77	Caecum	4.5 cm	Adenocarcinoma	Stage III
5	Male	43	Rectosigmoid	5.6 cm	Adenocarcinoma	Stage III

After transferring the tissues to the laboratory under sterile conditions, the samples were washed 3 times in Phosphate-buffered saline (PBS) containing 5% fetal bovine serum (FBS) and 3% penicillin/streptomycin antibiotics. Then, peripheral and necrotic tissues were removed, and the remaining tumor and normal tissues were fully cut up into 1 mm pieces and explanted in 6 well plates containing Dulbecco's modified Eagle's medium/Nutrient Mixture F-12 (DMEM/F12) (BI-1012, Bioidea, Iran) supplemented with 10% FBS (BI-1201, Bioidea, Iran), 1% penicillin–streptomycin (100X, BI-1203, Bioidea, Iran), and 2 mmol/L L-glutamine (BI-1202, Bioidea, Iran). Fibroblast-like cells were let to migrate out of the tissue fragments for 15–20 days. Once the cultured cells reach a > 70–80% confluent monolayer, harvested with 25% trypsin–EDTA and transferred to T25 flasks for further expansion.

### Co-culture of fibroblast and *F. nucleatum* components

The co-culture experiments were carried out in conditions in which primary fibroblasts were adapted as described above. Briefly, fibroblast cells (passage 3) were seeded at 1 × 10^5^ cells per well in 6-well plates containing 2 mL DMEM) without L-glutamine and cultured for 48 h in a humidified incubator at 37 °C and 5% of CO2. After that, cells were washed twice with PBS and new media containing the different components of the *F. nucleatum*, including secretome and ghost was added to infect the monolayer cells*.* There were 4 conditions for each extracted NF and CAF, including the cells without treatment as the control group, the cells treated with secretome in a 1:1 ratio in medium, the cells treated with the ghost of heat-killed bacteria, and the cells treated with the ghost of PFA-fixed bacteria. The *F. nucleatum* ghosts were added to the cells at a multiplicity of infection (MOI) of 100 and incubated for more than 48 h.

### Flow cytometry analysis

After encountering CAF and NF cells with *F. nucleatum* fragments, the cells were harvested, re-suspended in PBS, and washed twice. To perform surface staining for FAP marker detection, cold PBS containing the mouse anti-human FAP antibody (1: 40, cat#FAB3715, R&D system, USA) was added. After 30 min of incubation at 4 ºC, the cells were washed with cold PBS. Then, cells were fixed with 4% formaldehyde for 15 min, permeabilized with cold 1 × Perm/wash (cat#421002, Biolegend, USA) for 30 min, and incubated with mouse anti-human α-SMA antibody (1: 20, R&D system, cat#ICI420P, USA) for 40 min in the dark. This was followed by centrifugation and cells were resuspended in PBS. All stained cells were analyzed by flow cytometry (BD Bioscience, USA). Analysis of the obtained results was performed using FlowJo v10.8 (FlowJo LLC, USA).

### Enzyme-linked immunosorbent assay (ELISA)

After 48-h incubation of CAF and NF cells with *F. nucleatum* components in 6-well plates, fibroblast-conditioned media was collected and stored at −80 ºC for cytokine assay. Human IL-6 (Cat. 430,504, Biolegend, USA) and human TGF-β (Karmania Pars Gene, Iran), were assessed using ELISA according to the manufacturer’s protocol. Each sample was assayed in triplicate. Concentration was quantified by measuring the absorption at 450 nm with a microplate reader.

### Quantification of *F. nucleatum* in *colon* tissue by real-time PCR

After surgical resection, the colorectal tumor and adjacent nonmalignant tissue samples were snap-frozen in liquid nitrogen and stored at −70 °C. Genomic DNA was extracted from the tissues using the phenol–chloroform method [44].

To quantify the amount of *F. nucleatum* DNA in each sample, real-time PCR was performed for the *F. nucleatum nusG* gene. The cycle threshold (Ct) values for *F. nucleatum* were normalized to the amount of human biopsy gDNA in each reaction by using the reference gene, prostaglandin transporter (PGT), too.

qPCR was performed on the ABI Step One Real-time PCR instrument (Applied Biosystems, USA).

Primers used for assays were as follows:

*F. nucleatum* nusG forward primer, 5'CAACCATTACTTTAACTCTACCATGTTCA-3'; *F. nucleatum* nusG reverse' primer, 5′-GTTGACTTTACAGAAGGAGATTATGTAAAAATC-3′; PGT forward primer, 5′-ATCCCC AAAGCACCTGGTTT-3′; PGT reverse primer, 5′-AGAGGCCAAGATAGTCCTGGTAA-3′;

For all assays, each reaction well contained 6 µl of master mix (RealQ Plus 2 × Master Mix Green High ROX (Amplicon, Denmark)) and 3 µl of tissue extracted total DNA (at 30 ng/µl), in a 12 µl reaction mixture containing, 0.3 µl (10 pmol) of forward and reverse primers. The PCR conditions were 95 °C for 10 min followed by 45 cycles of 95 °C for 15 s, and 60 °C for 1 min. Mean cycle threshold (Ct) values were normalized by calculating ΔCt using the reference gene PGT and calculating using the 2^-ΔΔCT^ method.

### Statistical analysis

Statistical analyses were performed using GraphPad Prism software version 9.4.1. Comparisons of differences between the means of two treatment groups were performed using the paired two-tailed student's t-test, while comparisons among multiple groups were carried out using one-way or two-way analysis of variance (ANOVA), followed by Tukey’s multiple comparisons. The results are expressed as the mean ± SD, and a P value < 0.05 was considered to be statistically significant.

## Results

### *F. nucleatum* isolation and characterization

After clinical evaluations of more than 60 cases, 16 patients with periodontitis who met the desired inclusion criteria were identified by the dentist and candidates for sampling. After bacterial culture, 11 samples were excluded from the study due to the mismatch of colony shape, microscopic appearance, and biochemical tests.

**Fig. 1:**
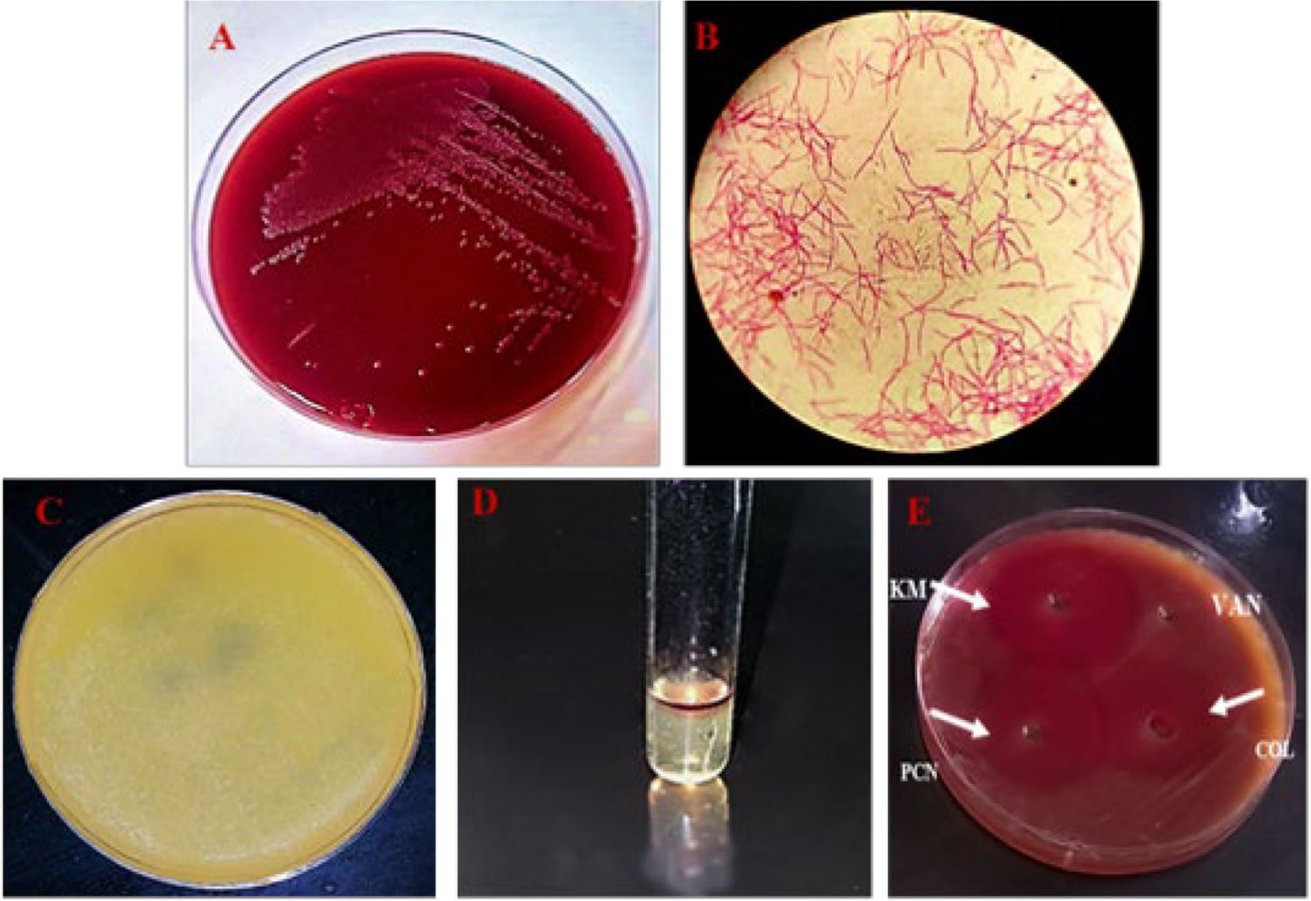
characterization of isolated *F. nucleatum*. **A** Dry, irregular, crumb-like circular colonies of *F. nucleatum* on Columbia agar plate. **B** *Morphological confirmation andgram staining of isolated bactria, F. nucleatum* is gram-negative with a long, thin, spindle-shaped appearance in gram staining, (× 100). C and D) biochemical characterization of isolated strain (**C**) The absence of a clear zone around bacterial colonies indicates the negativity of the lipase test, **D** the formation of a red ring on the SIM medium indicates the positivity of the indole test for isolated strain*,* **E** the presence of inhibition zones around kanamycin, penicillin, and colistin antibiotic indicates the sensitivity of *F. nucleatum* while the absence of inhibition zones around the vancomycin indicates the resistance of the *F. nucleatum* to it. *KM* kanamycin, *PCN* penicillin, *VAN* vancomycin, *COL* colistin

Isolates with white–gray speckled colonies that were dry, irregular, crumb-like circular with an entire edge on CA selective plate were presumed to be *F. nucleatum* (Fig. 1A)*.* Gram-staining for these colonies, confirmed the presence of gram-negative unique spindle shape with tapered ends species (Fig. 1B). The identification of the isolates at the species level was done using biochemical tests. The isolates that were lipase negative (the absence of clear zone around bacterial colonies), indole positive (the formation of a red ring on SIM medium), susceptible to kanamycin, penicillin, and colistin but resistant to vancomycin were considered as *F. nucleatum* (Fig. 1C–E).

Finally, bacterial ghosts (Heat treated and PFA-fixed) and secretomes were prepared as mentioned above.

### Normal and cancerous fibroblast cells out- growth and morphological characterization

**Fig. 2:**
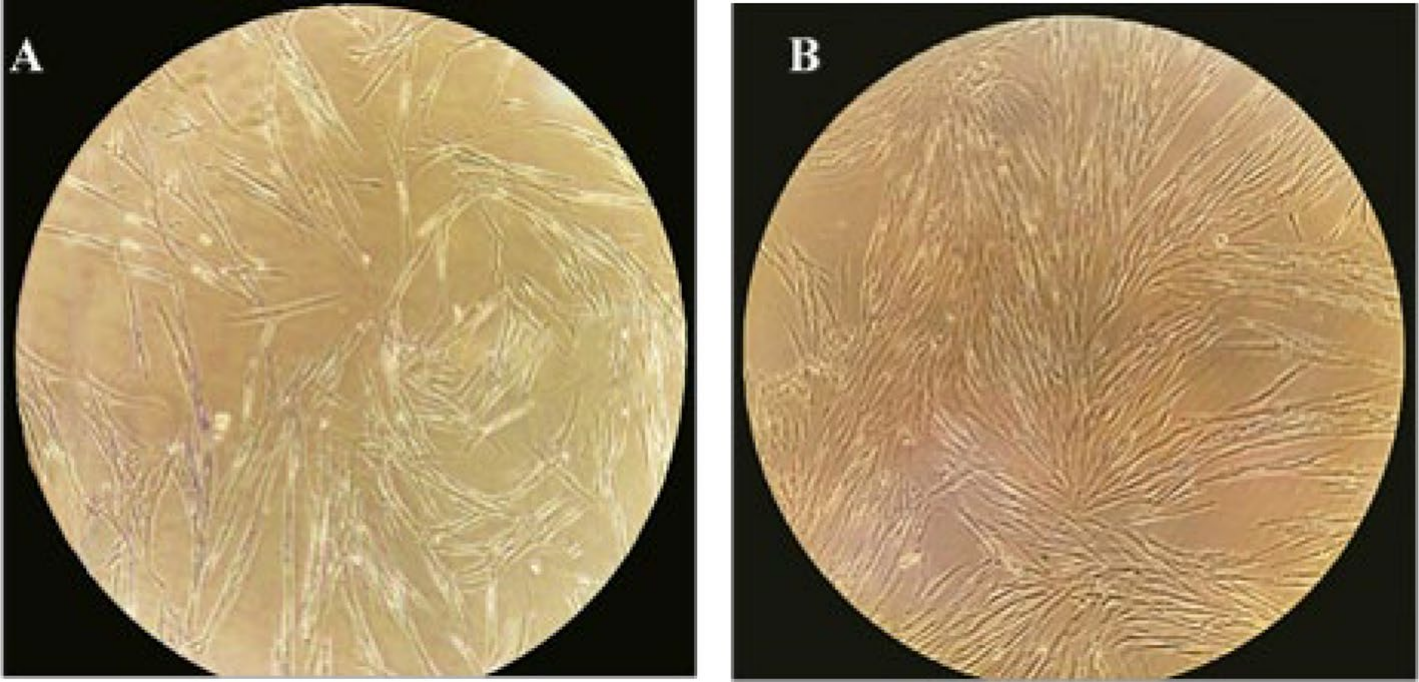
Morphological features of primary fibroblasts isolated from colon. **A** Cancer-associated fibroblast, and **B** normal fibroblast cells isolated from colon tissue in passage 3 (× 100)

Isolated fibroblast cells from normal and cancerous colorectal tissue by explant method, sub-cultured, and after reaching passages3-5, they were used for the next analysis. From the point of morphological view, the elongated, spindle-like shape of cells was observed using light microscopy to represent fibroblast-like cells. NFs are smaller, spindle-shaped, and homogeneous, while CAFs are stellate, with large and multiple nuclei and very granular (Fig. 2).

### FAP as a marker that differentiates CAFs from NFs

**Fig. 3:**
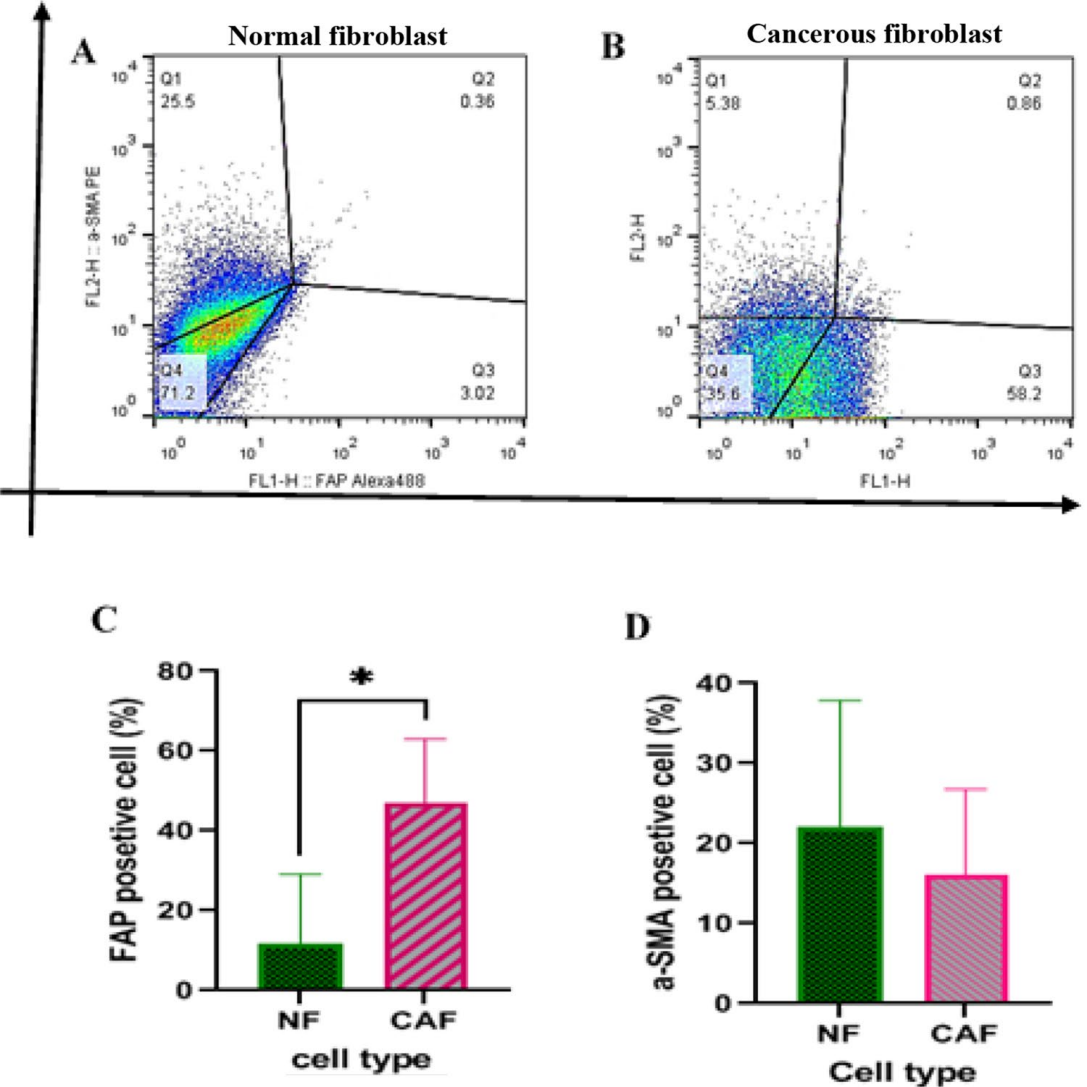
Normal and cancerous fibroblast isolated from colon tissue differentially expressed FAP and α-SMA. Representative flow cytometry data showed the frequency of α-SMA^+^ and FAP.^+^ fibroblastic cells isolated from normal **(A)** and cancerous colon tissue **(B)**. Comparison of the percentage of cells expressing FAP (**C**) and α-SMA (**D**) among normal and cancerous fibroblastic cells, (*p < 0.05)

To compare the expression pattern of FAP and α-SMA in NF and CAF, the flow cytometry analysis was performed. The percentage of cells expressing the FAP marker was significantly higher in the CAF cell population compared to NF cells (*P = 0.016, Fig. 3A, C). Although the expression pattern of the α-SMA marker was heterogeneous in both cells, it was higher in NF cells population than CAF cells; however, this difference was not statistically significant (Fig. 3B, D).

### Effect of *F. nucleatum* components on the expression pattern of FAP and α-SMA by fibroblast cells

To investigate the effect of *F. nucleatum* components on the fibroblast’s phenotype, we examined the expression patterns of FAP and α-SMA in different treatment conditions.

Considering the heterogeneity of the expression pattern of FAP and α-SMA markers in CAF and NF cells of different patients, the results of the effect of different treatments represented a relative fold change between treatment and control for each patient (Table 2).

**Table 2:** The percentage of NF and CAF cells expressing α-SMA and FAP markers in the groups treated with *F. nucleatum* components relative to the control group

sample	α-SMA in control group (%)	Heat/control ratio	PFA/control ratio	Secretome/control ratio	FAP in the control group (%)	Heat/control ratio	PFA/control ratio	Secretome/control ratio
Tumor 1	25.92	0.53	0.51	0.14	67.42	1.19	1.25	0.85
Tumor 2	15.3	1.43	1.26	0.49	44.6	0.71	1.06	0.66
Tumor 3	27.87	1.37	1.15	0.56	33.17	1.04	1.34	0.33
Tumor 4	6.24	0.52	0.60	1.05	59.06	0.65	0.80	0.37
Tumor 5	4.88	4.20	1.77	0.63	29.56	0.16	0.13	0.44
Normal 1	4.14	0.39	0.77	1.32	4.56	1.14	1.39	0.03
Normal 2	41.7	0.56	0.46	0.19	37.8	0.67	1.23	0.78
Normal 4	16.53	1.02	0.32	0.03	1.81	0.37	1.01	1.24
Normal 5	25.86	0.78	0.97	0.56	3.38	1.18	1.30	3.5

**Fig. 4:**
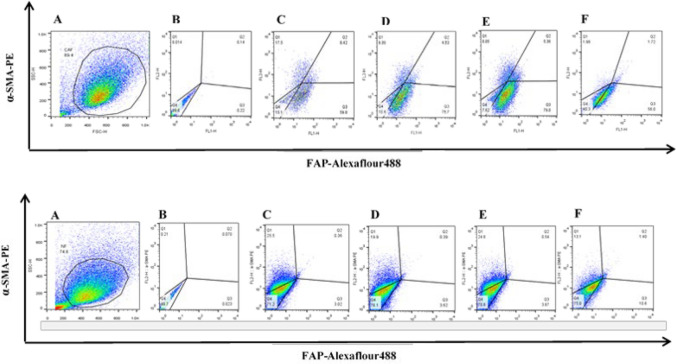
Effect of the *F. nucleatum* on the expression of FAP and α-SMA by fibroblast cells. Representative Flow cytometry data of fibroblasts from the tumor (top) and normal (bottom) tissue in coculture with *F. nucleatum* based on the expression of two markers, FAP and α-SMA**.** The gating of cells in an FSC vs. SSC plot to separate fibroblast cell populations based on size and granularity (**A**), unstained tube (**B**), cells expressing FAP and α-SMA markers in the control (untreated) group (**C**), the group treated with heat-killed ghosts (**D**), the group treated with PFA-fixed ghosts (**E**) and the group treated with secretome of *F. nucleatum.* (**F**)

The flow cytometry analysis showed that concerning the expression of the two mentioned markers followed by the treatment with different *F. nucleatum* components, isolated fibroblast obtained from normal and cancerous colon tissue, do not behave differently and in general, treatment with different bacterial components did not lead to significant changes in the expression of FAP and α-SMA markers (Fig. 4).

### Comparison of IL-6 and TGF-β levels in the supernatant of fibroblast cells before and after treatment with *F. nucleatum* components

To investigate the function of normal and cancerous fibroblast cells before and after treatment with *F. nucleatum* components, the concentration of IL-6 and TGF-β cytokines in CAFs and NFs conditioned media was assayed by ELISA.

**Fig. 5:**
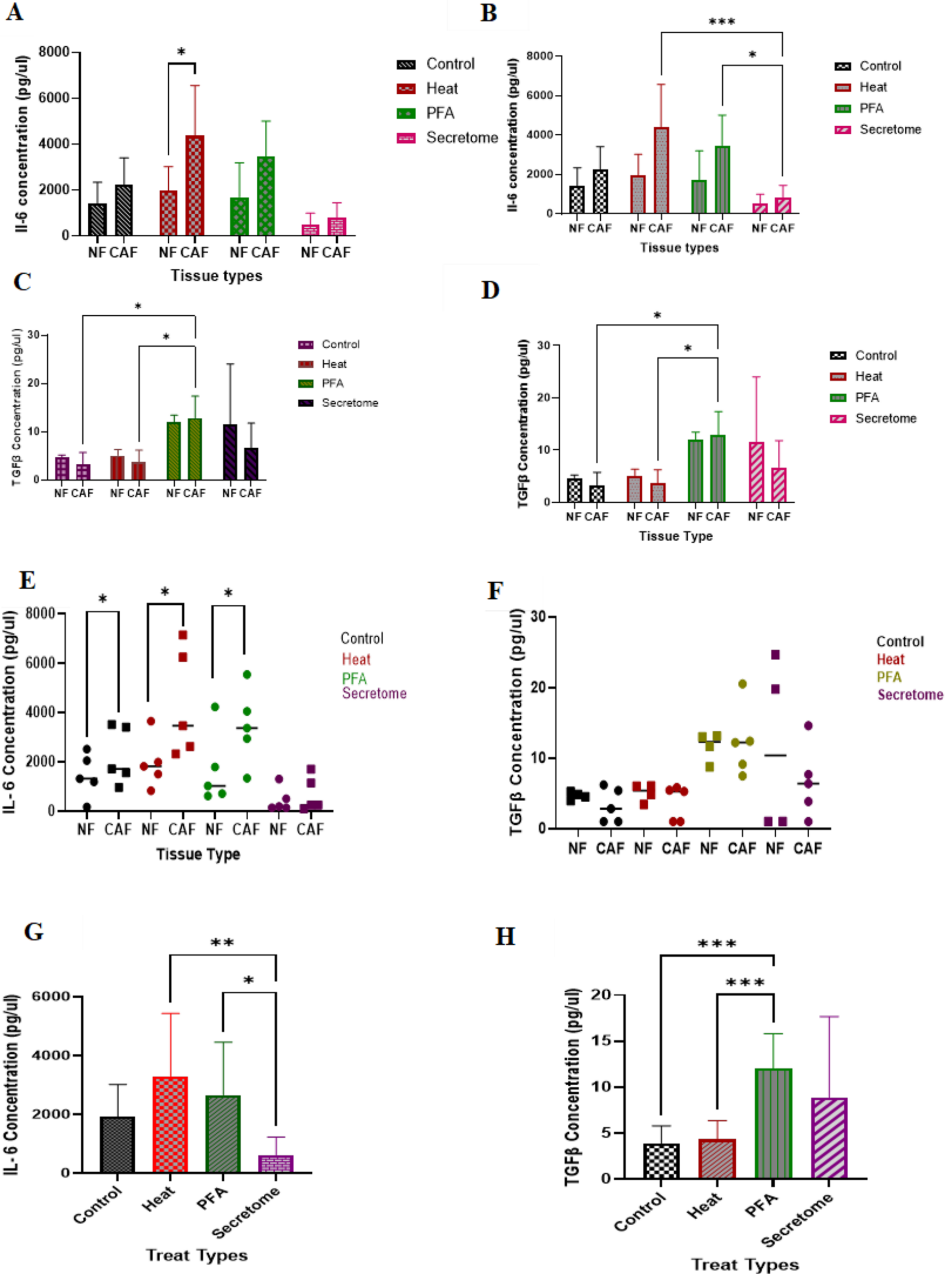
The expression of the IL-6 and TGF-β proteins was measured in supernatants from CAFs and NFs cells before and after treatment with *F. nucleatum* components using ELISA. Comparison of IL-6 (**A**) and TGF-β (**C**) produced by CAF and NF cells before and after treatment, comparison of IL-6 (**B**) and TGF-β (**D**) produced by CAF cells before and after treatment relative to each other and by NF cells before and after treatment relative to each other, comparison of IL-6 (**E**) and TGF-β (**F**) produced between each patient's NF cells with his CAF cells under 4 treatments using Paired T-Test, comparison of the effects of different treatments on the expression of cytokines IL-6 (**G**) and TGF-β (**H**) produced by fibroblastic cells using One-Way ANOVA. *p < 0.05, **p < 0.005, ***p < 0.0005

Our results showed that the amount of IL-6 in the supernatant of CAF cells before and after treatment in all 4 conditions (untreated, treated with heat-killed and PFA fixed ghosts and secretome) was higher than in NF cells. However, this difference was significant only in the group treated with the heated ghost (*p < 0.05, Fig. 5A).

Comparison of the amount of IL-6 in the 4 mentioned conditions, in the CAFs and NFs group separately showed that the treatment of CAF cells with ghosts significantly increased the secretion of IL-6 compared to the treatment with secretome-treated ones (*p < 0.05, ***p < 0.0005**,** Fig. 5B).

The amount of TGF-β in the supernatant of CAF cells before and after treatment in all 4 conditions was compared to NF cells, but no statistically significant difference was observed (Fig. 5C) between groups. However, significant overexpression of TGF-β was detected in CAF cells after exposure to PFA-fixed ghost in comparison with heated ghost and untreated groups (*p < 0.05, Fig. 5D).

Due to the heterogeneity between the results, in addition to comparing the average expression by the above methods, each patient's NFs were compared with his CAFs under 4 different conditions using the Paired T-Test. IL-6 levels were significantly higher in the treatment of CAF cells with *F. nucleatum* components except sectertome compared to NF cells (*p < 0.05**,** Fig. 5E). Also, no statistically significant difference was observed concerning TGF-β levels in this comparison (Fig. 5F).

Next, we used the One-Way ANOVA test to investigate the effect of different treatments in comparison with each other. In this case, all tumoral and normal tissues were considered as one group and were compared under four conditions. The results of this analysis showed that treatment with two types of ghosts increased the production of cytokine IL-6 compared to the untreated group, but none of them showed significant differences. However, this difference was significant between the groups treated with two types of ghosts compared to the group treated with secretions (*p < 0.05, **p < 0.005**,** Fig. 5G). Treatment with PFA-fixed ghost and secretions increased the levels of this TGF-β compared to the untreated group; however, the difference was statistically significant only between PFA-fixed ghost treated group compared to the untreated group. Also, the level of TGF-β was significantly higher in the PFA-fixed ghost-treated group compared to the heated ghost-treated group (**p < 0.005, ***p < 0.0005**,** Fig. 5H).

### Comparison of *F. nucleatum* presence in normal and tumor tissues

**Fig. 6:**
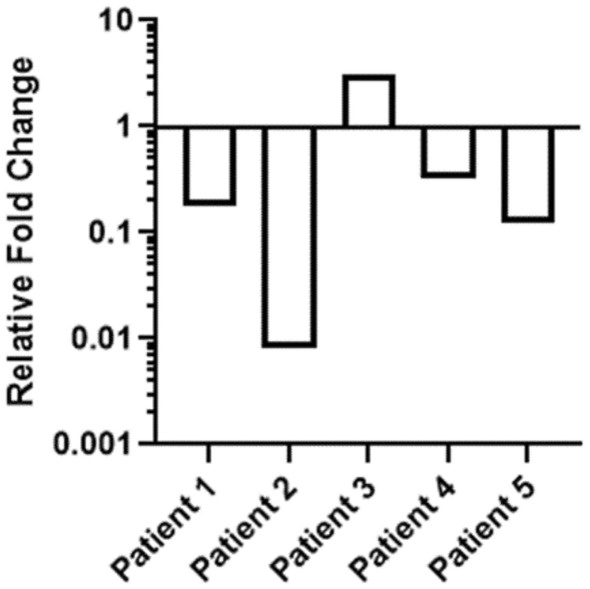
Relative quantification of the *F. nucleatum* in CRC and adjacent normal mucous tissues. The mean relative abundance of *F. nucleatum* (normalized to PGT expression) in CRC tissue versus adjacent normal tissue of both independent runs is reported for each patient sample. The y-axis represents mean fold gene expression change (2^−ΔΔCt^) while the x-axis represents patient samples

To reveal the previous exposure of both normal and tumoral tissues with *F. nucleatum*, qPCR was conducted. The analysis of the relative *nusG* gene expression in CRC tissues was conducted in a paired way by the adjacent normal tissue of each sample as its specific calibrator. Among the clinical samples with paired tumor and normal samples, the relative amount of the *nusG* gene was lower in tumor tissue than in adjacent normal tissue in 4 patients (Fig. 6) but in one patient it was higher in tumor tissue than in adjacent normal tissue.

## Discussion

CAFs, as the most crucial and abundant cells in the stroma of solid tumors such as CRC or PDAC are in reciprocal communication with TME components and as a result affects the growth and progression of tumors. Moreover, with the deep study of the interaction mechanism between CAFs and immune cells in the TME, a growing amount of evidence indicates CAFs leading immune cells polarization towards activation or exhaustion. In this mutual interaction, both CAFs and tumor cells are capable of altering each other’s functions [10]. So, to target CAFs for optimizing tumor therapy, identifying the characteristics of CAFs as well as their functions in the TME is of great importance. Also, identifying the factors that lead to the transformation of NFs into CAFs and the aggressiveness of this cell has a key role, too.

From another point of view, CAFs are known to comprise a diverse cell population consisting of several subtypes [45]. Support for this notion comes from recent studies on a variety of cancers, such as PDAC, breast or colon carcinoma [46], oral carcinoma [47], and lung cancer [48], in which functionally distinct subclasses of CAFs were identified by the expression of a limited set of markers [49]. Among these markers, α-SMA and FAP are among the most important markers of fibroblast cells, which are involved in a wide range of CAFs activities, including tumor growth and proliferation, inhibition of the immune system, prevention of drug delivery, metastasis, and angiogenesis. Also, available data confirmed FAP is the main marker of CAF activity in CRC [50].

FAP is one of the most strongly expressed genes in the stroma of solid tumors and is upregulated in over 90% of epithelial carcinomas. Due to its high expression in the tumor stroma, numerous studies have used FAP as a marker of activated CAFs [51–54].

This has resulted in the widespread use of FAP as an identifier of potential CAF populations, typically in combination with other markers [55]. FAP is also widely considered one of the most viable CAF markers for potential clinical application. Depletion of the FAP-positive fibroblast population in transgenic mice led to cytokine-mediated hypoxic necrosis of both the tumor and the stroma [28]. For this reason, FAP-based therapies, such as FAP-inhibitors and FAP-targeting monoclonal antibodies, have been submitted for clinical trials these days. It has also been shown that targeting FAP using CAR-T cells can effectively increase the killing of tumor cells in vitro [56]. Jingjing et al. showed that the cause of desmoplastic TME formation in CRC is the interaction between FAP + CAFs and SPP1 + macrophages, which results in resistance to immunotherapy and further reduces the efficacy of PD-L1 treatment [57]. Thus, there is interest in FAP + CAFs as a potential target for anti-tumor treatments, and existing research suggests that FAP-targeted drugs can exert curative effects in models of most solid tumors [29]. Moreover, several studies have shown the significance of α-SMA-positive CAFs in development and progression of solid tumors [58].

In line with the above, one of the main goals of this study was to compare the expression pattern of two markers α-SMA and FAP in CAF and NF cells isolated from colon tissue of patients with CRC. Our results showed that the expression pattern of these markers is different in isolated CAF and NF cells. The expression of α-SMA in normal and cancerous fibroblasts was not significantly different, although in NF it had a higher expression. But the expression of the FAP marker was significantly higher in the CAF cells.

Consistent with our findings other studies concerning the expression pattern of two mentioned markers, α-SMA and FAP, by fibroblast subsets provide similar results. For example, in women with a high prevalence of ductal carcinoma breast cancer, CAFs make up 50 to 70% of the cells in the TME. In this tumor, FAP overexpression was typically observed in the connective tissue, the main location of CAF cells, which led to the conclusion that FAP could be considered a major marker for CAFs. However, normal breast tissue has low and sometimes undetectable levels of FAP expression [29, 59]. In another study conducted by Yashiro et al., the percentage of NF and CAF cells expressing α-SMA isolated from stomach tissue was estimated at 42% and 18%, respectively, confirming the higher expression of α-SMA marker in NF cells than in CAF cells [60]. However, according to the expression intensity, FAP^Hi^ and α-SMA^Hi^ fibroblasts, respectively form different populations of CAF and NF cells in tumors derived from epithelial cells [61].

Another important finding of our study was the heterogeneous expression pattern of the two mentioned markers among different patients. So, the percentage of FAP^+^ cells in the CAF population varied from 29 to 67% and in NF cells from 2 to 37%. Also, the percentage of α-SMA^+^ cells in CAF cells of patients varied from 4 to 28% and in NF cells from 4 to 42%. To confirm this finding, we refer to the study on 217 patients with CRC conducted by Mo Son et al. in 2019, that showed the percentage of FAP + CAF and α-SMA + CAFs in patients varied from 0% (in 4 patients) to98% and 23%, respectively [62]. Also, during another study in 2020, 92 CRC tissues and 19 normal tissues adjacent to the tumor tissue were examined in terms of FAP marker expression. Eight tumor tissues and four normal tissues were negative in terms of FAP expression, and 84 tumor tissues and 15 normal tissues expressed the FAP marker, of which 72 tumor tissues and 1 normal tissue showed a high level of expression of the FAP marker at the surface of CAFs [61]. Therefore, according to the available data, although FAP can be a marker for targeting CAFs and as a result TME, due to its heterogeneous expression, FAP targeting in tumors should be based on the expression pattern of this marker.

After conducting various studies, it was found that a part of this heterogeneity is explained by the fact that CAFs originate from different cells [63, 64]. Another part of the heterogeneity of CAFs is related to their different function in TME. So, some CAFs secrete some growth factors such as FGF, IL-6, and IL-8, which cause the growth and expansion of cancer cells and the polarization of immune cells towards exhausted cells. Meanwhile, in the context of drug resistance, it is approved that CAFs activate the JAK-STAT3 and PI3K-Akt pathways in cancer cells by secreting IL-6, inducing the up-regulation of epithelial − mesenchymal transition (EMT) and E3 ubiquitin ligase complex function [30, 65].

The secretome analysis of separated fibroblasts in our study showed the expression of IL-6 by CAF cells was higher than that of NF cells, but TGF-β produced in both groups of CAF and NF cells was low and did not differ significantly from each other. In this regard, Ohlund and his colleagues reported that CAFs with a low expression level of the α-SMA marker produce a large amount of IL-6, and CAFs that express a high amount of FAP and α-SMA produce more TGF-β that both subtypes play an important role in tumor progression [66]. Another study on pancreatic cancer showed that α-SMA^+^ CAF cells produce macrophage colony-stimulating factor 1 (M-CSF-1), IL-6, TGF-β, and CCL2 to promote monocyte recruitment and macrophage polarization into M2 macrophages [24].

Microbiome plays a significant role in the complex media of CRC microenvironment and its critical role in the development and progression of the tumor and immune system function has been indicated [67].

One of the most important components of the microbiome is *F. nucleatum*. Various studies have shown that cell surface and intracellular proteins, and microbial and cell secretions of this bacteria, which include exosomes, growth factors, cytokines, and secreted DAMPs can affect the TME [66, 68, 69]. Also, specifically, the role of secretions and proteins of *F. nucleatum* has been proven in tumor formation and development [70]. In this regard, one of the goals of this study was to investigate the effect of *F. nucleatum* components on the function and phenotype of fibroblast cells. For this purpose, the effect of bacterial secretions and ghosts on the percentage of α-SMA and FAP and IL-6 and TGF-β production were evaluated.

Following the treatment of CAfs and NFs to the heat-killed ghosts, the expression of the α-SMA marker in CAF cells was often increased compared to the control group, although it was not significant. However, this increasing pattern was not observed in NF cells. Therefore, it can be assumed that the killing of bacteria and the spilling out of proteins and internal bacterial components such as genome and LPS have an effect on increasing the expression of α-SMA marker in CAF cells, which can potentially affect the inhibitory functions of CAFs [40, 68, 71–74].

The treatment of CAF and NF cells with PFA-fixed ghosts also showed similar results for α-SMA and FAP markers. This over-expression could be due to the exposure to bacterial surface pathogenic proteins such as FadA, RadD, and Fap2 that could be increased fibroblast activity to produce tumorigenic factors such as α-SMA and FAP markers. In the group treated with secretome, the expression of FAP and α-SMA markers often decreased compared to the control group, although it was not statistically significant, which is probably due to the death of fibroblast cells caused by the lethality of the secretome.

A study has shown during the killing of *F. nucleatum*, endotoxins, especially lipopolysaccharide (LPS) of the bacteria are released, which are recognized by TLRs on the surface of epithelial cells and fibroblasts. Also, the intracellular danger signals then lead to the activation of NLRP3 and the subsequent release of cytokines such as IL-1β [68]. *F. nucleatum* induces the production of inflammatory cytokines by activating NF-κB through TLR4 receptor signaling and by producing FadA protein that binds to E-cadherin of host epithelial and fibroblast cells, which activates the β-catenin signaling pathway. These functions play an important role in creating oncogenic and inflammatory responses and forming a suitable environment for tumor growth [40, 71–73, 75]. Also, a study has shown that a large number of bacterial virulence factors, which are effective in the initiation and progression of inflammatory bowel diseases, were present in the secretions of *F. nucleatum*, which can lead to tumorigenesis in host cells [76].

In all treatment conditions, the amount of IL-6 in the supernatant of CAF cells was higher than in NF cells, but it was significant only in the group treated with heated ghosts. The TGF-β expression in the supernatant of CAF and NF cells was not significantly different in any treatment. Also, after comparing the overall effect of the treatments with each other, we observed a significant increase in the production of IL-6 in the group treated with ghosts (heated and PFA-fixed) compared to the group treated with secretome.

About TGF-β, this increase was also observed in the group treated with PFA-fixed ghosts compared to the untreated group. Also, the level of TGF-β production was significantly higher in the group treated with PFA-fixed ghosts compared to the heated-ghost treated group.

In this regard, previous studies showed that the presence of *F. nucleatum* in the intestines of people with CRC stimulates the inflammatory response, which leads to the ROS production, the release of inflammatory factors such as COX-2, IL-1β, IL-6, IL-8, and TNFα from the cells of the TME, and epigenetic changes in cancer cells [42, 73]. In 2019, Wu et al. stated that *F. nucleatum* binds to intestinal epithelial cells and fibroblasts by its surface proteins, including FadA, Fap2, and RadD, and induces the migration of inflammatory cells and the production of inflammatory factors. In addition, *F. nucleatum* can inhibit the intestinal immune system by suppressing the function of immune cells such as macrophages, T cells, and NK cells and contribute to the development of CRC [70]. Li and his colleagues also have indicated *F. nucleatum* isolated from the mouth affects cancer cells in oral squamous cell cancer, causing them to produce IL6 and IL8. These cytokines affect CAFs and lead to the activation of the JAK/STAT pathway in CAF cells and ultimately induce the production of VEGF and tumor angiogenesis [66].

## Importance

According to the importance of *Fusobacterium nucleatum* as well as cancer associated fibroblasts in the induction and progression of colorectal cancer, this is the first study preliminary investigate regarding the effect of *F. nucleatum* on the function and phenotype of isolated fibroblast cells.

## Conclusion

According to our findings, α-SMA and FAP expression on CAF and NF is variable and probably influenced by TME condition. Also, the higher expression of FAP on the surface of CAF cells compared to NF cells besides the important role of FAP in tumor development and immune system suppression during other studies indicates the importance of this marker in tumor development, so that it can be considered as a therapeutic target in the tumor. However, due to its heterogeneous expression, FAP targeting in tumors should be based on the expression pattern of this marker. Furthermore, it should be noted that because FAP expresses on the surface of other cells such as macrophages, identification and targeting of other CAF-specific markers should be considered. In addition, due to the association of FAP with worse prognosis, invasion, metastasis, and treatment resistance in several cancers, the expression level of the FAP marker can be used as a biomarker in determining prognosis, recurrence, and diagnosis of tumor status.

Moreover, our study demonstrated that the interaction between F. nucleatum components and fibroblasts is crucial in modulating the inflammatory state in the TME. However, the limited sample size in our study is a constraint. Further detailed studies involving larger populations and more markers are required to explore the effects of this relation in the tumor milieu.

## Data Availability

The data that support the findings of this study are available from the corresponding author, upon reasonable request.

## References

[1] Arnold M, Sierra MS, Laversanne M, Soerjomataram I, Jemal A, Bray F. Global patterns and trends in colorectal cancer incidence and mortality. Gut. 2017;66(4):683–91. 10.1136/gutjnl-2015-310912. 26818619 10.1136/gutjnl-2015-310912

[2] Xi Y, Xu P. Global colorectal cancer burden in 2020 and projections to 2040. Trans Oncol. 2021;14(10): 101174. 10.1016/j.tranon.2021.101174. 10.1016/j.tranon.2021.101174 PMC8273208 34243011

[3] Bray F, Ferlay J, Soerjomataram I, Siegel RL, Torre LA, Jemal A. Global cancer statistics 2018: GLOBOCAN estimates of incidence and mortality worldwide for 36 cancers in 185 countries. CA A Cancer J Clini. 2018;68(6):394–424. 10.3322/caac.21492. 10.3322/caac.21492 30207593

[4] Lee JB, Kim K-A, Cho HY, Kim D, Kim WK, Yong D, et al. Association between Fusobacterium nucleatum and patient prognosis in metastatic colon cancer. Sci Rep. 2021;11(1):20263. 10.1038/s41598-021-98941-6. 34642332 10.1038/s41598-021-98941-6 PMC8511250

[5] Giraldo NA, Sanchez-Salas R, Peske JD, Vano Y, Becht E, Petitprez F, et al. The clinical role of the TME in solid cancer. Br J Cancer. 2019;120(1):45–53. 10.1038/s41416-018-0327-z. 30413828 10.1038/s41416-018-0327-z PMC6325164

[6] Eskandari-Malayeri F, Rezaei M. Immune checkpoint inhibitors as mediators for immunosuppression by cancer-associated fibroblasts: a comprehensive review. Front Immunol. 2022;13: 996145. 10.3389/fimmu.2022.996145. 36275750 10.3389/fimmu.2022.996145 PMC9581325

[7] Sahai E, Astsaturov I, Cukierman E, DeNardo DG, Egeblad M, Evans RM, et al. A framework for advancing our understanding of cancer-associated fibroblasts. Nat Rev Cancer. 2020;20(3):174–86. 10.1038/s41568-019-0238-1. 31980749 10.1038/s41568-019-0238-1 PMC7046529

[8] Monteran L, Erez N. The dark side of fibroblasts: cancer-associated fibroblasts as mediators of immunosuppression in the tumor microenvironment. Front Immunol. 2019. 10.3389/fimmu.2019.01835. 31428105 10.3389/fimmu.2019.01835 PMC6688105

[9] Zhou W, Xu G, Wang Y, Xu Z, Liu X, Xu X, et al. Oxidative stress induced autophagy in cancer associated fibroblast enhances proliferation and metabolism of colorectal cancer cells. Cell Cycle. 2017;16(1):73–81. 10.1080/15384101.2016.1252882. 27841696 10.1080/15384101.2016.1252882 PMC5270538

[10] Chen P-Y, Wei W-F, Wu H-Z, Fan L-S, Wang W. Cancer-associated fibroblast heterogeneity: a factor that cannot be ignored in immune microenvironment remodeling. Front Immunol. 2021;12: 671595. 10.3389/fimmu.2021.671595. 34305902 10.3389/fimmu.2021.671595 PMC8297463

[11] Herrera M, Berral-González A, López-Cade I, Galindo-Pumariño C, Bueno-Fortes S, Martín-Merino M, et al. Cancer-associated fibroblast-derived gene signatures determine prognosis in colon cancer patients. Mol Cancer. 2021;20(1):73. 10.1186/s12943-021-01367-x. 33926453 10.1186/s12943-021-01367-x PMC8082938

[12] Kennel KB, Bozlar M, De Valk AF, Greten FR. Cancer-associated fibroblasts in inflammation and antitumor immunity. Clin Cancer Res. 2023;29(6):1009–16. 10.1158/1078-0432.CCR-22-1031. 36399325 10.1158/1078-0432.CCR-22-1031 PMC10011884

[13] Geng X, Chen H, Zhao L, Hu J, Yang W, Li G, et al. Cancer-associated fibroblast (CAF) heterogeneity and targeting therapy of CAFs in pancreatic cancer. Front Cell Dev Biol. 2021;9: 655152. 10.3389/fcell.2021.655152. 34336821 10.3389/fcell.2021.655152 PMC8319605

[14] Deng L, Jiang N, Zeng J, Wang Y, Cui H. The versatile roles of cancer-associated fibroblasts in colorectal cancer and therapeutic implications. Front Cell Dev Biol. 2021. 10.3389/fcell.2021.733270. 34660589 10.3389/fcell.2021.733270 PMC8517274

[15] Jena BC, Sarkar S, Rout L, Mandal M. The transformation of cancer-associated fibroblasts: current perspectives on the role of TGF-β in CAF mediated tumor progression and therapeutic resistance. Cancer Lett. 2021;520:222–32. 10.1016/j.canlet.2021.08.002. 34363903 10.1016/j.canlet.2021.08.002

[16] Monteran L, Erez N. The dark side of fibroblasts: cancer-associated fibroblasts as mediators of immunosuppression in the tumor microenvironment. Front Immunol. 2019;10:1835. 10.3389/fimmu.2019.01835. 31428105 10.3389/fimmu.2019.01835 PMC6688105

[17] Wu X, Tao P, Zhou Q, Li J, Yu Z, Wang X, et al. IL-6 secreted by cancer-associated fibroblasts promotes epithelial-mesenchymal transition and metastasis of gastric cancer via JAK2/STAT3 signaling pathway. Oncotarget. 2017;8(13):20741. 10.18632/oncotarget.15119. 28186964 10.18632/oncotarget.15119 PMC5400541

[18] Shintani Y, Fujiwara A, Kimura T, Kawamura T, Funaki S, Minami M, et al. IL-6 secreted from cancer-associated fibroblasts mediates chemoresistance in NSCLC by increasing epithelial-mesenchymal transition signaling. J Thorac Oncol. 2016;11(9):1482–92. 10.1016/j.jtho.2016.05.025. 27287412 10.1016/j.jtho.2016.05.025

[19] Mao X, Xu J, Wang W, Liang C, Hua J, Liu J, et al. Crosstalk between cancer-associated fibroblasts and immune cells in the tumor microenvironment: new findings and future perspectives. Mol Cancer. 2021;20(1):1–30. 10.1186/s12943-021-01428-1. 34635121 10.1186/s12943-021-01428-1 PMC8504100

[20] Ahmadzadeh M, Rosenberg SA. TGF-β1 attenuates the acquisition and expression of effector function by tumor antigen-specific human memory CD8 T cells. J Immunol. 2005;174(9):5215–23. 10.4049/jimmunol.174.9.5215. 15843517 10.4049/jimmunol.174.9.5215 PMC2562293

[21] Feig C, Jones JO, Kraman M, Wells RJ, Deonarine A, Chan DS, et al. Targeting CXCL12 from FAP-expressing carcinoma-associated fibroblasts synergizes with anti–PD-L1 immunotherapy in pancreatic cancer. Proc Natl Acad Sci. 2013;110(50):20212–7. 10.1073/pnas.1320318110. 24277834 10.1073/pnas.1320318110 PMC3864274

[22] Miyai Y, Esaki N, Takahashi M, Enomoto A. Cancer-associated fibroblasts that restrain cancer progression: hypotheses and perspectives. Cancer Sci. 2020;111(4):1047–57. 10.1111/cas.14346. 32060987 10.1111/cas.14346 PMC7156845

[23] Özdemir BC, Pentcheva-Hoang T, Carstens JL, Zheng X, Wu C-C, Simpson TR, et al. Depletion of carcinoma-associated fibroblasts and fibrosis induces immunosuppression and accelerates pancreas cancer with reduced survival. Cancer Cell. 2014;25(6):719–34. 10.1016/j.ccr.2014.04.005. 24856586 10.1016/j.ccr.2014.04.005 PMC4180632

[24] Liu T, Han C, Wang S, Fang P, Ma Z, Xu L, et al. Cancer-associated fibroblasts: an emerging target of anti-cancer immunotherapy. J Hematol Oncol. 2019;12(1):1–15. 10.1186/s13045-019-0770-1. 31462327 10.1186/s13045-019-0770-1 PMC6714445

[25] Kadel D, Zhang Y, Sun H-R, Zhao Y, Dong Q-Z, Qin L-x. Current perspectives of cancer-associated fibroblast in therapeutic resistance: potential mechanism and future strategy. Cell Biol Toxicol. 2019. 10.1007/s10565-019-09461-z. 30680600 10.1007/s10565-019-09461-z PMC6881418

[26] Kobayashi H, Enomoto A, Woods SL, Burt AD, Takahashi M, Worthley DL. Cancer-associated fibroblasts in gastrointestinal cancer. Nat Rev Gastroenterol Hepatol. 2019;16(5):282–95. 10.1038/s41575-019-0115-0. 30778141 10.1038/s41575-019-0115-0

[27] Yang X, Lin Y, Shi Y, Li B, Liu W, Yin W, et al. FAP promotes immunosuppression by cancer-associated fibroblasts in the tumor microenvironment via STAT3–CCL2 SignalingFAP via STAT3–CCL2 promote tumor immunosuppression. Can Res. 2016;76(14):4124–35. 10.1158/0008-5472.CAN-15-2973. 10.1158/0008-5472.CAN-15-2973 27216177

[28] Kraman M, Bambrough PJ, Arnold JN, Roberts EW, Magiera L, Jones JO, et al. Suppression of antitumor immunity by stromal cells expressing fibroblast activation protein–α. Science. 2010;330(6005):827–30. 10.1126/science.1195300. 21051638 10.1126/science.1195300

[29] Xin L, Gao J, Zheng Z, Chen Y, Lv S, Zhao Z, et al. Fibroblast activation protein-α as a target in the bench-to-bedside diagnosis and treatment of tumors: a narrative review. Front Oncol. 2021. 10.3389/fonc.2021.648187. 34490078 10.3389/fonc.2021.648187 PMC8416977

[30] Sun X, Mao Y, Wang J, Zu L, Hao M, Cheng G, et al. IL-6 secreted by cancer-associated fibroblasts induces tamoxifen resistance in luminal breast cancer. Oncogene. 2014. 10.1038/onc.2014.158. 24909173 10.1038/onc.2014.158

[31] Joshi RS, Kanugula SS, Sudhir S, Pereira MP, Jain S, Aghi MK. The role of cancer-associated fibroblasts in tumor progression. Cancers. 2021. 10.3390/cancers13061399. 33808627 10.3390/cancers13061399 PMC8003545

[32] Mezawa Y, Orimo A. The roles of tumor-and metastasis-promoting carcinoma-associated fibroblasts in human carcinomas. Cell Tissue Res. 2016;365(3):675–89. 10.1007/s00441-016-2471-1. 27506216 10.1007/s00441-016-2471-1

[33] Gascard P, Tlsty TD. Carcinoma-associated fibroblasts: orchestrating the composition of malignancy. Genes Dev. 2016;30(9):1002–19. 10.1101/gad.279737.116. 27151975 10.1101/gad.279737.116 PMC4863733

[34] Surowiak P, Murawa D, Materna V, Maciejczyk A, Pudelko M, Ciesla S, et al. Occurence of stromal myofibroblasts in the invasive ductal breast cancer tissue is an unfavourable prognostic factor. Anticancer Res. 2007;27(4C):2917–24. 10.1007/s00428-002-0639-4. 17695471 10.1007/s00428-002-0639-4

[35] Tsujino T, Seshimo I, Yamamoto H, Ngan CY, Ezumi K, Takemasa I, et al. Stromal myofibroblasts predict disease recurrence for colorectal cancer. Clin Cancer Res. 2007;13(7):2082–90. 10.1158/1078-0432.CCR-06-2191. 17404090 10.1158/1078-0432.CCR-06-2191

[36] Hanley CJ, Noble F, Ward M, Bullock M, Drifka C, Mellone M, et al. A subset of myofibroblastic cancer-associated fibroblasts regulate collagen fiber elongation, which is prognostic in multiple cancers. Oncotarget. 2016;7(5):6159. 10.18632/oncotarget.6740. 26716418 10.18632/oncotarget.6740 PMC4868747

[37] Temraz S, Nassar F, Nasr R, Charafeddine M, Mukherji D, Shamseddine A. Gut microbiome: a promising biomarker for immunotherapy in colorectal cancer. Int J Mol Sci. 2019;20(17):4155. 10.3390/ijms20174155. 31450712 10.3390/ijms20174155 PMC6747470

[38] Ranjbar M, Salehi R, Haghjooy Javanmard S, Rafiee L, Faraji H, Jafarpor S, et al. The dysbiosis signature of Fusobacterium nucleatum in colorectal cancer-cause or consequences? a systematic review. Cancer Cell Int. 2021;21(1):194. 10.1186/s12935-021-01886-z. 33823861 10.1186/s12935-021-01886-z PMC8025348

[39] Fong W, Li Q, Yu J. Gut microbiota modulation: a novel strategy for prevention and treatment of colorectal cancer. Oncogene. 2020. 10.1038/s41388-020-1341-1. 32514151 10.1038/s41388-020-1341-1 PMC7314664

[40] Koi M, Okita Y, Carethers JM. Fusobacterium nucleatum infection in colorectal cancer: linking inflammation, DNA mismatch repair and genetic and epigenetic alterations. J Anus Rectum Colon. 2018;2(2):37–46. 10.23922/jarc.2017-055. 30116794 10.23922/jarc.2017-055 PMC6090547

[41] Kang W, Jia Z, Tang D, Zhang Z, Gao H, He K, et al. Fusobacterium nucleatum facilitates apoptosis, ROS generation, and inflammatory cytokine production by activating AKT/MAPK and NF-κB signaling pathways in human gingival fibroblasts. Oxidative Med Cell Longevity. 2019. 10.1155/2019/1681972. 10.1155/2019/1681972 PMC6815639 31737164

[42] Zhou Z, Chen J, Yao H, Hu H. Fusobacterium and colorectal cancer. Front Oncol. 2018;8:371. 10.3389/fonc.2018.00371. 30374420 10.3389/fonc.2018.00371 PMC6196248

[43] Aliramezani A, Salari MH, Pourmand MR, Kadkhoda Z, Foroushani A, Aminharati F, et al. Prevalence of periodontopathogenic bacteria in patients suffering from periodontitis using culture and PCR methods. J Dental Med. 2012;25(3):159–65.

[44] Wilson T, Stark C, Holmbom J, Rosling A, Kuusilehto A, Tirri T, et al. Fate of bone marrow-derived stromal cells after intraperitoneal infusion or implantation into femoral bone defects in the host animal. J Tissue Eng. 2010;1(1): 345806. 10.4061/2010/345806. 10.4061/2010/345806 PMC3042670 21350643

[45] Louault K, Li R-R, DeClerck YA. Cancer-associated fibroblasts: understanding their heterogeneity. Cancers. 2020;12(11):3108. 10.3390/cancers12113108. 33114328 10.3390/cancers12113108 PMC7690906

[46] Li H, Courtois ET, Sengupta D, Tan Y, Chen KH, Goh JJL, et al. Reference component analysis of single-cell transcriptomes elucidates cellular heterogeneity in human colorectal tumors. Nat Genet. 2017;49(5):708–18. 10.1038/ng.3818. 28319088 10.1038/ng.3818

[47] Patel AK, Vipparthi K, Thatikonda V, Arun I, Bhattacharjee S, Sharan R, et al. A subtype of cancer-associated fibroblasts with lower expression of alpha-smooth muscle actin suppresses stemness through BMP4 in oral carcinoma. Oncogenesis. 2018;7(10):78. 10.1038/s41389-018-0087-x. 30287850 10.1038/s41389-018-0087-x PMC6172238

[48] Su S, Chen J, Yao H, Liu J, Yu S, Lao L, et al. CD10+ GPR77+ cancer-associated fibroblasts promote cancer formation and chemoresistance by sustaining cancer stemness. Cell. 2018;172(4):841–56. 10.1016/j.cell.2018.01.009. 29395328 10.1016/j.cell.2018.01.009

[49] Nurmik M, Ullmann P, Rodriguez F, Haan S, Letellier E. In search of definitions: cancer-associated fibroblasts and their markers. Int J Cancer. 2020;146(4):895–905. 10.1002/ijc.32193. 30734283 10.1002/ijc.32193 PMC6972582

[50] Avery D, Govindaraju P, Jacob M, Todd L, Monslow J, Puré E. Extracellular matrix directs phenotypic heterogeneity of activated fibroblasts. Matrix Biol. 2018;67:90–106. 10.1016/j.matbio.2017.12.003. 29248556 10.1016/j.matbio.2017.12.003 PMC5910258

[51] Berdiel-Acer M, Sanz-Pamplona R, Calon A, Cuadras D, Berenguer A, Sanjuan X, et al. Differences between CAFs and their paired NCF from adjacent colonic mucosa reveal functional heterogeneity of CAFs, providing prognostic information. Mol Oncol. 2014;8(7):1290–305. 10.1016/j.molonc.2014.04.006. 24839936 10.1016/j.molonc.2014.04.006 PMC5528579

[52] De Marco P, Lappano R, Francesco EMD, Cirillo F, Pupo M, Avino S, et al. GPER signalling in both cancer-associated fibroblasts and breast cancer cells mediates a feedforward IL1β/IL1R1 response. Sci Rep. 2016;6(1):24354. 10.1038/srep24354. 27072893 10.1038/srep24354 PMC4829876

[53] Kramer N, Schmöllerl J, Unger C, Nivarthi H, Rudisch A, Unterleuthner D, et al. Autocrine WNT2 signaling in fibroblasts promotes colorectal cancer progression. Oncogene. 2017;36(39):5460–72. 10.1038/onc.2017.144. 28553956 10.1038/onc.2017.144

[54] Isella C, Terrasi A, Bellomo SE, Petti C, Galatola G, Muratore A, et al. Stromal contribution to the colorectal cancer transcriptome. Nat Genet. 2015;47(4):312–9. 10.1038/ng.3224. 25706627 10.1038/ng.3224

[55] Strating E, Verhagen MP, Wensink E, Dünnebach E, Wijler L, Aranguren I, et al. Co-cultures of colon cancer cells and cancer-associated fibroblasts recapitulate the aggressive features of mesenchymal-like colon cancer. Front Immunol. 2023;14:1053920. 10.3389/fimmu.2023.1053920. 37261365 10.3389/fimmu.2023.1053920 PMC10228738

[56] Bughda R, Dimou P, D’Souza RR, Klampatsa A. Fibroblast activation protein (FAP)-targeted CAR-T cells: launching an attack on Tumor stroma. Immunotargets Ther. 2021;10:313–23. 10.2147/itt.s291767. 34386436 10.2147/itt.s291767 PMC8354246

[57] Qi J, Sun H, Zhang Y, Wang Z, Xun Z, Li Z, et al. Single-cell and spatial analysis reveal interaction of FAP+ fibroblasts and SPP1+ macrophages in colorectal cancer. Nat Commun. 2022;13(1):1742. 10.1038/s41467-022-29366-6. 35365629 10.1038/s41467-022-29366-6 PMC8976074

[58] Muchlińska A, Nagel A, Popęda M, Szade J, Niemira M, Zieliński J, et al. Alpha-smooth muscle actin-positive cancer-associated fibroblasts secreting osteopontin promote growth of luminal breast cancer. Cell Mol Biol Lett. 2022;27(1):45. 10.1186/s11658-022-00351-7. 35690734 10.1186/s11658-022-00351-7 PMC9188043

[59] Hua X, Yu L, Huang X, Liao Z, Xian Q. Expression and role of fibroblast activation protein-alpha in microinvasive breast carcinoma. Diagn Pathol. 2011;6(1):111. 10.1186/1746-1596-6-111. 22067528 10.1186/1746-1596-6-111 PMC3228672

[60] Liu T, Han C, Wang S, Fang P, Ma Z, Xu L, et al. Cancer-associated fibroblasts: an emerging target of anti-cancer immunotherapy. J Hematol Oncol. 2019;12(1):86. 10.1186/s13045-019-0770-1. 31462327 10.1186/s13045-019-0770-1 PMC6714445

[61] Coto-Llerena M, Ercan C, Kancherla V, Taha-Mehlitz S, Eppenberger-Castori S, Soysal SD, et al. High expression of FAP in colorectal cancer is associated with angiogenesis and immunoregulation processes. Front Oncol. 2020;10:979. 10.3389/fonc.2020.00979. 32733792 10.3389/fonc.2020.00979 PMC7362758

[62] Son GM, Kwon M-S, Shin D-H, Shin N, Ryu D, Kang C-D. Comparisons of cancer-associated fibroblasts in the intratumoral stroma and invasive front in colorectal cancer. Medicine. 2019;98(18): e15164. 10.1097/MD.0000000000015164. 31045759 10.1097/MD.0000000000015164 PMC6504275

[63] Yoon H, Tang C-M, Banerjee S, Delgado AL, Yebra M, Davis J, et al. TGF-β1-mediated transition of resident fibroblasts to cancer-associated fibroblasts promotes cancer metastasis in gastrointestinal stromal tumor. Oncogenesis. 2021;10(2):13. 10.1038/s41389-021-00302-5. 33568624 10.1038/s41389-021-00302-5 PMC7876107

[64] Wang J, Zhang G, Wang J, Wang L, Huang X, Cheng Y. The role of cancer-associated fibroblasts in esophageal cancer. J Transl Med. 2016;14:30. 10.1186/s12967-016-0788-x. 26822225 10.1186/s12967-016-0788-x PMC4732002

[65] Dana P, Thumrongsiri N, Tanyapanyachon P, Chonniyom W, Punnakitikashem P, Saengkrit N. Resveratrol loaded liposomes disrupt cancer associated fibroblast communications within the tumor microenvironment to inhibit colorectal cancer aggressiveness. Nanomaterials. 2022;13(1):107. 10.3390/nano13010107. 36616017 10.3390/nano13010107 PMC9824711

[66] Musa M, Ali A. Cancer-associated fibroblasts of colorectal cancer and their markers: updates, challenges and translational outlook. Future Oncol. 2020;16(29):2329–44. 10.2217/fon-2020-0384. 32687721 10.2217/fon-2020-0384

[67] Rebersek M. Gut microbiome and its role in colorectal cancer. BMC Cancer. 2021;21(1):1325. 10.1186/s12885-021-09054-2. 34895176 10.1186/s12885-021-09054-2 PMC8666072

[68] Chen Y, Huang Z, Tang Z, Huang Y, Huang M, Liu H, et al. More Than Just a periodontal pathogen-the research progress on Fusobacterium nucleatum. Front Cell Infect Microbiol. 2022;12: 815318. 10.3389/fcimb.2022.815318. 35186795 10.3389/fcimb.2022.815318 PMC8851061

[69] Peng L, Wang D, Han Y, Huang T, He X, Wang J, et al. Emerging role of cancer-associated fibroblasts-derived exosomes in tumorigenesis. Front Immunol. 2022;12:5661. 10.3389/fimmu.2021.795372. 10.3389/fimmu.2021.795372 PMC8764452 35058933

[70] Wu J, Li Q, Fu X. Fusobacterium nucleatum contributes to the carcinogenesis of colorectal cancer by inducing inflammation and suppressing host immunity. Trans Oncol. 2019;12(6):846–51. 10.1016/j.tranon.2019.03.003. 10.1016/j.tranon.2019.03.003 PMC6462820 30986689

[71] Keku TO, Dulal S, Deveaux A, Jovov B, Han X. The gastrointestinal microbiota and colorectal cancer. Am J Physiol Gastrointestinal Liver Physiol. 2015. 10.1152/ajpgi.00360.2012. 10.1152/ajpgi.00360.2012 PMC4346754 25540232

[72] Wang S, Liu Y, Li J, Zhao L, Yan W, Lin B, et al. Fusobacterium nucleatum acts as a pro-carcinogenic bacterium in colorectal cancer: from association to causality. Front Cell Dev Biol. 2021;9: 710165. 10.3389/fcell.2021.710165. 34490259 10.3389/fcell.2021.710165 PMC8417943

[73] Kang W, Jia Z, Tang D, Zhang Z, Gao H, He K, et al. Fusobacterium nucleatum facilitates apoptosis, ROS generation, and inflammatory cytokine production by activating AKT/MAPK and NF-κB signaling pathways in human gingival fibroblasts. Oxid Med Cell Longev. 2019;2019:1681972. 10.1155/2019/1681972. 31737164 10.1155/2019/1681972 PMC6815639

[74] Kang W, Ji X, Zhang X, Tang D, Feng Q. Persistent exposure to Fusobacterium nucleatum triggers chemokine/cytokine release and inhibits the proliferation and osteogenic differentiation capabilities of human gingiva-derived mesenchymal stem cells. Front Cell Infect Microbiol. 2019;9:429. 10.3389/fcimb.2019.00429. 31921705 10.3389/fcimb.2019.00429 PMC6927917

[75] Kang W, Ji X, Zhang X, Tang D, Feng Q. Persistent exposure to Fusobacterium nucleatum triggers chemokine/cytokine release and inhibits the proliferation and osteogenic differentiation capabilities of human gingiva-derived mesenchymal stem cells. Front Cell Infect Microbiol. 2019. 10.3389/fcimb.2019.00429. 31921705 10.3389/fcimb.2019.00429 PMC6927917

[76] Zanzoni A, Spinelli L, Braham S, Brun C. Perturbed human sub-networks by Fusobacterium nucleatum candidate virulence proteins. Microbiome. 2017;5(1):89. 10.1186/s40168-017-0307-1. 28793925 10.1186/s40168-017-0307-1 PMC5551000

